# Transcranial direct current stimulation of the prefrontal cortex modulates working memory performance: combined behavioural and electrophysiological evidence

**DOI:** 10.1186/1471-2202-12-2

**Published:** 2011-01-06

**Authors:** Tino Zaehle, Pascale Sandmann, Jeremy D Thorne, Lutz Jäncke, Christoph S Herrmann

**Affiliations:** 1 Department of Neurology, Otto v. Guericke University Magdeburg, Germany; 2 German Centre for Neurodegenerative Diseases (DZNE) Magdeburg, Germany; 3 Department of Psychology, Neuropsychology Lab, Carl von Ossietzky University of Oldenburg, Germany; 4 Institute of Psychology, Division of Neuropsychology, University of Zurich, Switzerland; 5 Department of Experimental Psychology, Carl von Ossietzky University Oldenburg, Germany

## Abstract

**Background:**

Transcranial direct current stimulation (tDCS) is a technique that can systematically modify behaviour by inducing changes in the underlying brain function. In order to better understand the neuromodulatory effect of tDCS, the present study examined the impact of tDCS on performance in a working memory (WM) task and its underlying neural activity. In two experimental sessions, participants performed a letter two-back WM task after sham and either anodal or cathodal tDCS over the left dorsolateral prefrontal cortex (DLPFC).

**Results:**

Results showed that tDCS modulated WM performance by altering the underlying oscillatory brain activity in a polarity-specific way. We observed an increase in WM performance and amplified oscillatory power in the theta and alpha bands after anodal tDCS whereas cathodal tDCS interfered with WM performance and decreased oscillatory power in the theta and alpha bands under posterior electrode sides.

**Conclusions:**

The present study demonstrates that tDCS can alter WM performance by modulating the underlying neural oscillations. This result can be considered an important step towards a better understanding of the mechanisms involved in tDCS-induced modulations of WM performance, which is of particular importance, given the proposal to use electrical brain stimulation for the therapeutic treatment of memory deficits in clinical settings.

## Background

Transcranial direct current stimulation (tDCS) is a technique that stimulates the cerebral cortex with a weak constant electric current in a non-invasive and painless manner [1]. The current flows from an active to a reference electrode, a part being shunted through the scalp and the rest being delivered to the brain tissue [2], thereby inducing diminutions or enhancements of cortical excitability [1]. The direction of the tDCS-induced effect depends on the current polarity: Anodal tDCS typically has an excitatory effect on the local cerebral cortex, while cathodal tDCS decreases the cortical excitability in the region under the electrode [3,4]. The mechanisms underlying these neuromodulatory effects are not well understood [5]. Animal studies suggest that anodal tDCS, via an extracellular negative sink, causes a depolarization of the resting-membrane potential and increases the firing rates of many perpendicularly oriented cortical neurons in the tissue under the electrode. Cathodal stimulation has the opposite effect, causing a hyperpolarisation of the resting-membrane potential and a decrease in firing rates [6,7]. Thus, tDCS seems to modify spontaneous neural excitability by tonic de- or hyperpolarization of the resting-membrane potential [1]. However, the effects of tDCS are not limited to modulations in cortical excitability during stimulation, and may outlast the stimulation period by several minutes or even hours [3,4,6,8]. These after effects of tDCS are associated with a number of different mechanisms, including local changes in ionic concentrations (hydrogen, calcium) and levels of cyclic adenosine monophosphate (cAMP), alterations in protein synthesis, and modulation of *N*-methyl-D-aspartate (NMDA) receptor efficacy [5,9-14].

The neuromodulatory changes induced by tDCS have been associated with modifications of a variety of behavioural brain functions. In animal studies, anodal tDCS of the cortical surface has been linked with facilitation of an unconditioned response [15,16] and improved learning [17]. In humans, the effects of tDCS have been demonstrated on various motor, visual, and somatosensory cortex functions (see [18] for a recent review). In particular, previous studies have reported enhancements in motor [19,20] and visuo-motor learning [21] for anodal tDCS, while impairments in auditory learning have been observed for cathodal tDCS [22]. Similarly, anodal tDCS improves language learning [23], picture-naming [24] as well as implicit grammar learning [25], whereas cathodal tDCS has been shown to impair verbal learning abilities [26,27]. Analogous polarity-specific effects of tDCS have been reported for working memory (WM) functions, suggesting that anodal but not cathodal tDCS can improve WM performance [8,28,29]. However, the nature of the neurophysiological mechanisms underlying this cognitive enhancement is not yet well understood, because modifications of WM functions by tDCS have never been studied in combination with neurophysiological methods.

In general, WM refers to a set of basic mental operations that define the ability to hold an item of information transiently in mind, in order to recall, manipulate and associate this information to incoming new information [30]. WM is crucial to many higher-order strategic functions and has been linked to frontal [31,32] and parietal lobe functioning [33]. A commonly used WM paradigm is the n-back task which activates a fronto-parietal network, including the dorsolateral prefrontal cortex (DLPFC) [34-37], and the posterior parietal cortex [38]. While the DLPFC is involved in the processing of stimulus information during retention times [39], the parietal lobe participates in the storage of perceptual attributes [40]. Furthermore, the prefrontal cortex seems to be functionally lateralized, with the right hemisphere being recruited in particular during spatial WM tasks, and the left hemisphere being crucial for the processing of non-spatial (i.e., verbal) WM tasks [41]. The critical role for the left DLPFC in verbal WM performance has been confirmed by lesion studies and studies using TMS, showing that focal damage and temporary disruption of the left but not the right DLPFC is related to impairments in verbal WM task performance [42,43].

The present study examined the impact of tDCS on WM performance and the underlying neural activity. In particular, we explored the effect of tDCS applied over the left DLPFC on oscillatory brain activity during a letter n-back WM task. Based on previous findings we hypothesized tDCS-dependent alteration of WM performance [8,29]. Furthermore, we predicted tDCS-related modifications of the underlying rhythmic neural activity in the alpha and theta frequency range, given the view that alpha and theta oscillations play an important role in memory functions [44]. To our knowledge this is the first study investigating the modulatory effects of tDCS on oscillatory brain activity in the context of a WM task. The better understanding of the neuromodulatory effects of tDCS is also of clinical interest, since electrical brain stimulation seems to have potential as a therapeutic tool applied for several neurological and psychiatric disorders [45-51], and particularly for the treatment of memory deficits in stroke patients [52], patients with Parkinson's disease [28], and patients suffering from Alzheimer's disease [53,54].

## Results

### Behavioural data

**Figure 1:**
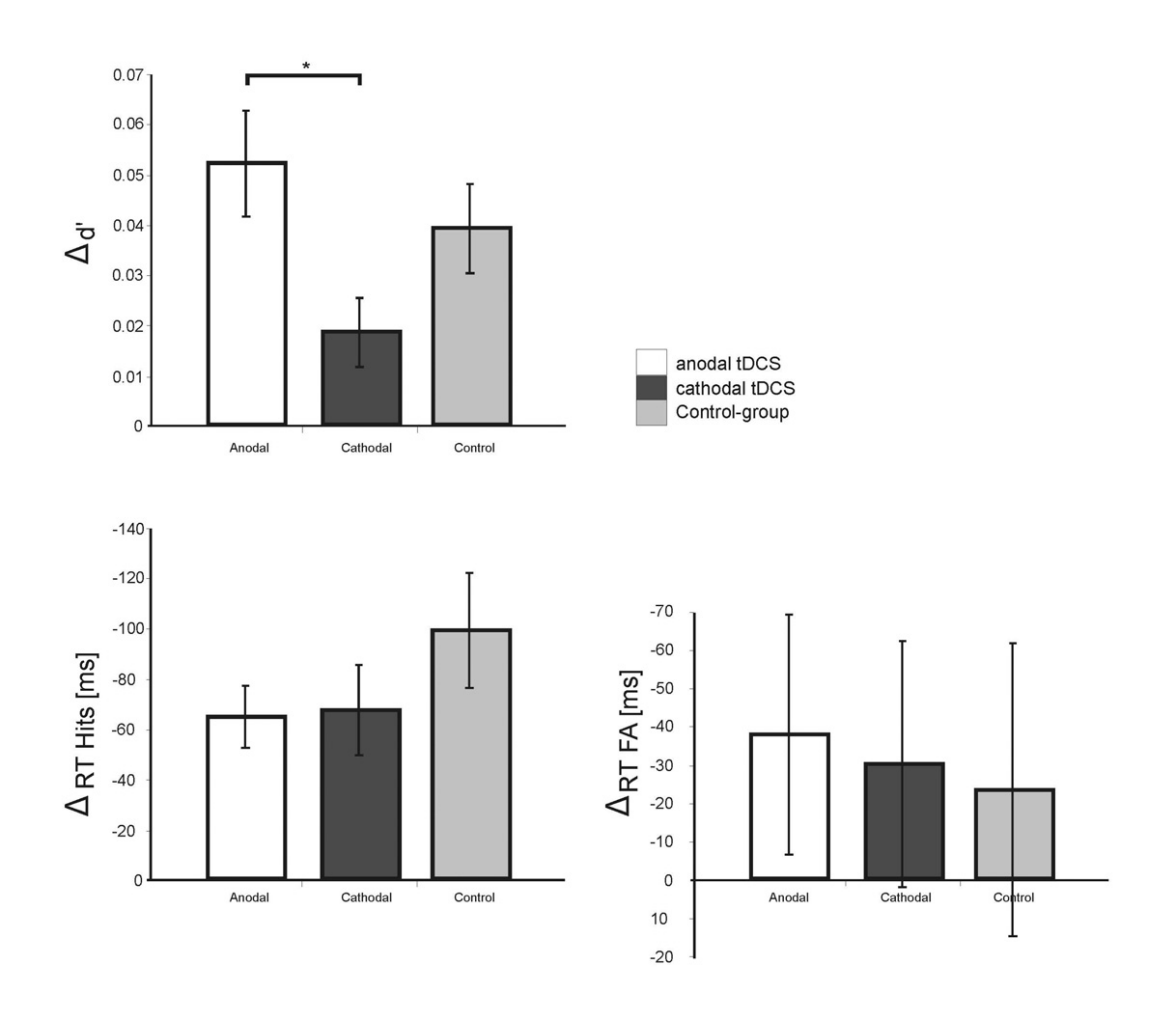
**Behavioural Data**: Plots show performance in the WM task after anodal and cathodal tDCS in relation to preceding sham stimulation as well as the performance of the separate control group. Top: The d' increased more after anodal than after cathodal stimulation with an intermediate effect for the control group, demonstrating a polarity effect of the tDCS-induced behavioural improvement. Bottom: Participants responded generally faster from sham to tDCS measure and pre-to-post measure in the control group for Hits (left) and False Alarms (right), respectively. Asterisks indicate statistical significance (*P *< 0.05).

Pre-to-post measurements revealed that all participants improved their performance during the experiment. Table 1 shows the results of one-sample t-tests separately for the tDCS-treated group in each stimulation condition (delta anodal **Δ**A, delta cathodal **Δ**C) and for the control group (delta **Δ**CG). The behavioural improvement from sham to active tDCS was stronger after anodal than after cathodal stimulation (cf. Figure 1). This polarity effect was statistically significant for the comparison of **Δ**A-d' >**Δ**C-d' (*t *_15 _= 2.14, *P *< 0.05). Analysis of reaction times (RT) showed that tDCS-treated participants responded faster after active tDCS compared to sham for *Hits*, but not for *False Alarms *(cf. Table 1). Similarly, pre-to-post measurements for the control group showed faster RT for *Hits *but not for *False Alarms*. Interestingly, the decrease of RT in the tDCS-treated group did not differ between anodal and cathodal stimulation (**Δ**A = **Δ**C), neither for *Hits *(*t*_15 _= 0.11, *P *= 0.9) nor for *False Alarms *(*t*_15 _= -0.13, *P *= 0.9).

**Table 1:** Improvement in performance

condition	measure	*t*	df	*P*
**Δ**A	d'	4.748	15	0.000
	RT-Hits	-5.370	15	0.000
	RT-FA	-1.210	15	0.244
**Δ**C	d'	2.751	15	0.015
	RT-Hits	-3.760	15	0.002
	RT-FA	-0.945	15	0.359
**Δ**CG	d'	3.944	15	0.001
	RT-Hits	-4.289	15	0.001
	RT-FA	-0.600	15	0.555

Table lists result of one-sample t-tests for d', RT-Hits (reaction time for Hits), and RT-FA (reaction time for False Alarms). Results show a general improvement in behaviour from pre- to post measurements for anodal (ΔA) and cathodal stimulation (ΔC), and in the separate control group (ΔCG).

In order to separate tDCS-induced alterations of WM performance from repetition-related learning effects, we compared the behavioural pre-to-post measurements between the tDCS-treated group and the control group (cf. Figure 1). The comparison revealed for the tDCS-treated group a stronger, but statistically non-significant d' increase after anodal stimulation (**Δ**A-d' >**Δ**CG-d', *t*_30 _= 0.99, *P *= 0.33), and a weaker d' increase after cathodal stimulation (**Δ**C-d' <**Δ**CG-d', *t*_30 _= -1.39, *P *= 0.17) compared to the control group. Analysis of RT showed that pre to post decreases did not differ between the control group and the tDCS-treated group for the anodal or cathodal session, either for *Hits *(**Δ**A = **Δ**CG: *t*_30 _= 1.29, *P *= 0.2, **Δ**C = **Δ**CG: *t*_30 _= 1.1, *P *= 0.3) or for *False Alarms *(**Δ**A = **Δ**CG: *t*_30 _= -0.3, *P *= 0.8, **Δ**C = **Δ**CG: *t*_30 _= -0.14, *P *= 0.9).

### Event-related potentials (ERPs)

**Figure 2:**
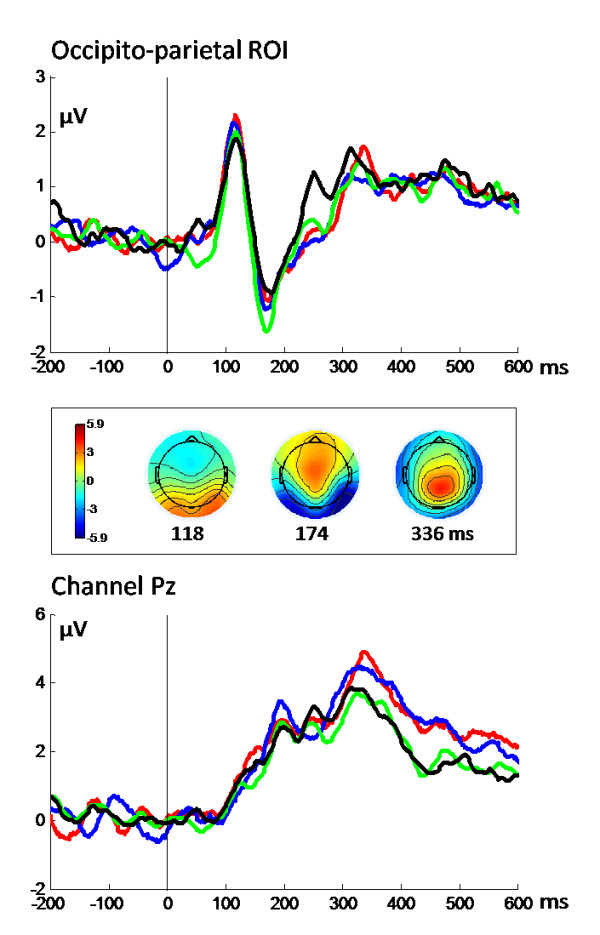
**Event-related potentials**: Grand average event-related potentials (ERPs) are shown for each condition at the occipito-parietal ROI (above) and at channel Pz (below). Topographies of overall grand averages (computed across all participants and conditions) are shown at P1, N1, and P3 latencies.

Figure 2 illustrates the event-related potential (ERP) data for anodal, cathodal and the two sham conditions (shamA, shamC) averaged over 16 subjects for the occipito-parietal region of interest (ROI) and electrode Pz. Visual stimulation consistently evoked a P1 component at 118 ms which was followed by the N1 component at 174 ms. A P3 component was elicited consistently in all tDCS conditions with a mean latency of 336 ms. Paired t-tests were performed to statistically compare the active and corresponding sham conditions (anodal vs. shamA, cathodal vs. shamC), and the anodal and cathodal conditions (shamA vs. shamC, anodal vs. cathodal). Statistical analyses of ERP amplitudes revealed no significant differences between conditions for the P1, N1 and P3 components. Regarding latencies, no significant differences were found between the two sham conditions. However, N1 latencies were significantly longer for the anodal than for the cathodal (*t*_15 _= 2.58, *P *< 0.05) and for the anodal compared to the shamA condition (*t*_15 _= -2.28, *P *< 0.05), and P3 latencies were significantly shorter for the cathodal compared to the shamC condition (*t*_15 _= 2.24, *P *< 0.05).

### Event-related spectral perturbation (ERSP)

**Figure 3:**
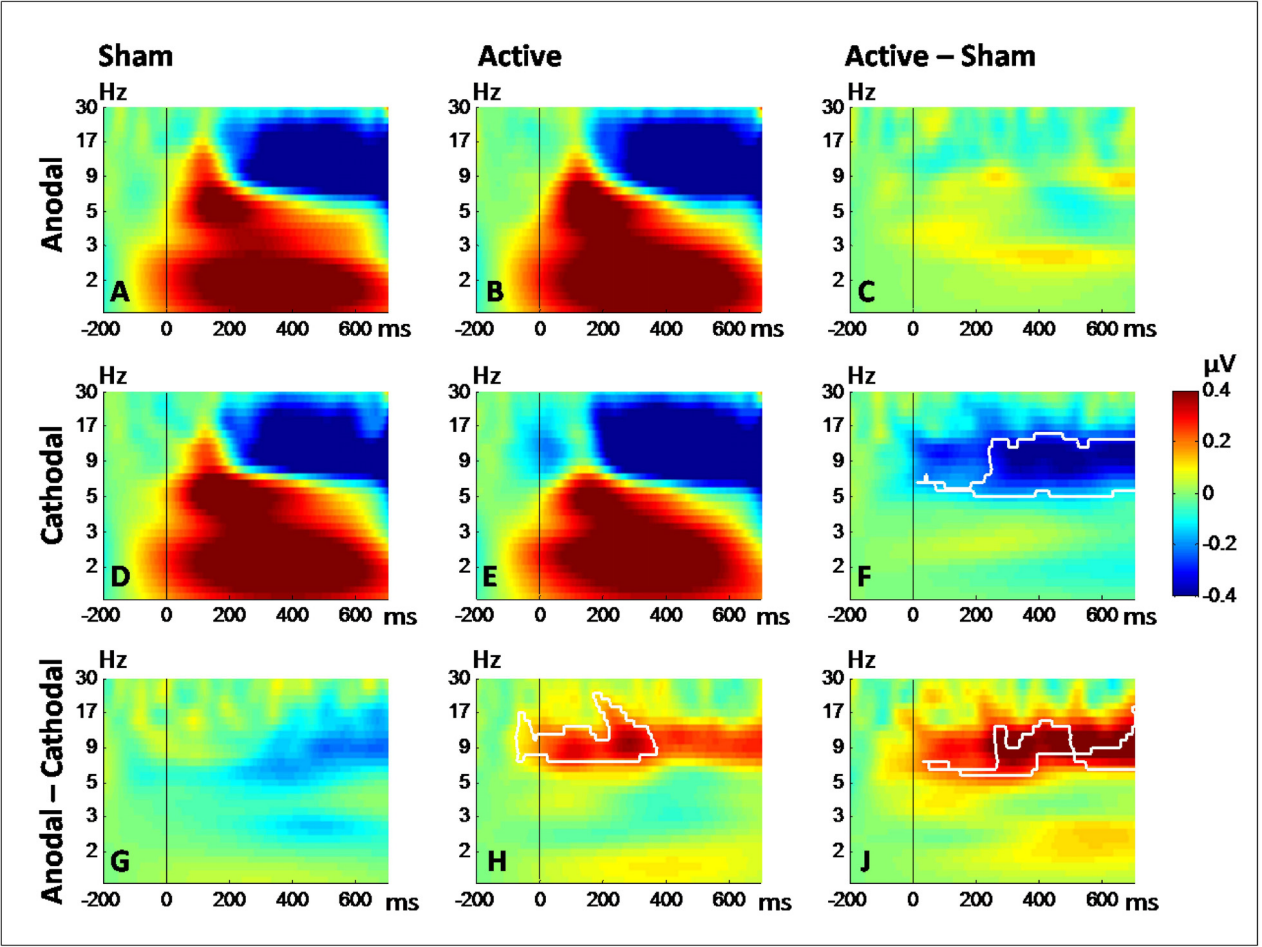
**DC stimulation effect on oscillatory brain activity**: Event-related spectral perturbation (ERSP) time-frequency plots are given separately for the two sham conditions (A: sham anodal; D: sham cathodal) preceding the two active stimulation conditions (B: anodal, E: cathodal). Differences between the conditions were computed by subtracting the sham ERSPs from the ERSPs of the active conditions (C: active anodal - sham anodal; F: active cathodal - sham cathodal) and by subtracting the cathodal ERSPs from anodal ERSPs (G: sham anodal - sham cathodal; H: active anodal - active cathodal). Subplot J illustrates the overall contrast between the differences obtained for the anodal and cathodal conditions (subplot C - subplot F) and for the active and sham conditions (subplot G - subplot H). White contours indicate significant differences between the conditions (*P *< 0.05, corrected for multiple comparisons). Note that the statistical analyses revealed no significant differences between the two sham conditions.

Figure 3 shows the event-related spectral perturbation (ERSP) time-frequency plots for the sham conditions (shamA, shamC) and the active stimulation conditions (anodal, cathodal), plus the corresponding differences at the occipito-parietal ROI. Statistical comparison of ERSPs between the shamC and cathodal stimulation condition revealed a decrease in the theta and alpha band (5 - 15 Hz), at the latency range of 14 - 710 ms (*t*_15 _≥ 2.13, *P *< 0.05). Anodal tDCS as compared to cathodal tDCS increased the total power in the theta, alpha, and lower beta band (7-24 Hz) at the latency range of -72 - 380 ms (*t*_15 _≥ 2.13, *P *< 0.05). Finally, a significant cluster (*t*_15 _≥ 2.13, *P *< 0.05) was found for the overall differences between sham and stimulation conditions ((anodal - shamA) vs. (cathodal - shamC)). This cluster had a latency range of -34 - 710 ms and revealed a significant increase in the theta, alpha, and lower beta band (6 - 19 Hz) for the anodal (anodal - shamA) compared to the cathodal session (cathodal - shamC).

## Discussion

We assessed the impact of transcranial direct current stimulation (tDCS) on working memory (WM) performance and the underlying neural activity. In two experimental sessions participants performed a letter two-back WM task after sham and either anodal or cathodal tDCS. The results showed that WM performance was generally enhanced from the sham to the active stimulation condition, indicating a repetition-related learning effect on WM task performance. Importantly, this regular improvement in WM performance was influenced by active tDCS in a polarity-specific way. The improvement in WM performance was significantly stronger after the application of anodal than after cathodal tDCS over the left dorsolateral prefrontal cortex (DLPFC), while the WM improvement was intermediate when participants received no electrical stimulation. Thus, anodal tDCS improved the regular repetition-related increase in WM performance, whereas cathodal tDCS interfered with this effect. These tDCS-induced effects were reflected in the neural oscillatory activity, showing polarity-specific alterations as a function of tDCS. Anodal tDCS increased whereas cathodal tDCS decreased the event-related oscillatory power in the theta and alpha range. Our results suggest that tDCS altered WM performance by modulating the underlying oscillatory brain activity in the theta and alpha frequency bands. These results we consider an important step towards a better understanding of the mechanisms involved in the tDCS-induced modulations of WM performance, which is particularly relevant as electrical brain stimulation has been proposed as a useful therapeutic modality for the treatment of memory deficits in a clinical context [28,52]. More specifically, the present results of tDCS-induced modulations of neural oscillations in specific frequency bands suggests that the combination of tDCS and EEG might provide a useful approach for the investigation of the functional significance of several oscillatory bands in human cognition. Furthermore, our results may provide additional explanations for tDCS-related therapeutic effects in patients suffering from Parkinson's disease or Alzheimer' disease. Based on our findings we predict that beneficial effects of tDCS in these patients may be associated with modulations in oscillatory brain activity, in particular in the alpha and theta frequency range. In addition, our results may inspire future research on tDCS-related therapeutic applications, in particular with respect to pathologies that have been associated with alterations of oscillatory brain activity in specific frequency bands.

### Behavioural effects of tDCS

The effect of tDCS on WM performance is consistent with recent findings on the modulatory effects of tDCS on WM functions [8,28,29,52]. Similar to our results, anodal but not cathodal stimulation over the left DLPFC has been shown to increase performance in a sequential-letter WM task [8,29]. Likewise, a beneficial effect of anodal tDCS over the left DLPFC on WM has been observed in patients with Parkinson's disease [28], and in patients suffering from stroke [52]. Our study further revealed that the general decrease in response time (RT) from sham to active tDCS did not vary with tDCS polarity. Similar RT insensitivity to tDCS has been previously reported [8,28,29,52], although one former study has observed increased RT after both anodal and cathodal stimulation over fronto-cortical regions [55]. This discrepancy between behavioural findings might be explained by the fact that the latter study evaluated the effects of bilateral and intermittent tDCS, while our study investigated unilateral and continuous tDCS. Furthermore, the difference in results between the studies may be a consequence of differences in electrode size, position and applied current intensities, and duration of the washout period between the active tDCS sessions. Computational approaches using spherical [2,56] and realistic finite element head models [57] have demonstrated that the size, location and shape of the stimulating electrode can influence the electric field in the underlying brain, thereby affecting the level of modulation of tDCS. Overall, our behavioural results, even though not novel per se, provide further evidence for a modulatory capability of tDCS on WM performance. Our results confirm previous findings by showing that tDCS can reliably induce alterations in WM performance in a polarity-dependent manner. More specifically, our results suggest that repetition-related improvement in WM performance is elevated by anodal tDCS, but diminished by cathodal tDCS.

### Electrophysiological correlates of tDCS effects

In this study we assessed oscillatory brain activity during the WM task in order to investigate the underlying neural mechanisms mediating the tDCS-induced behavioural effects. To date, reports of electrophysiological correlates of tDCS effects are sparse. Using visual event-related potentials (ERPs), a previous study has demonstrated that the amplitude of the N70 ERP component is increased by anodal tDCS, while it is decreased by cathodal tDCS [58]. The opposite effect has been reported for the visual P100, showing reduced amplitudes for anodal and increased amplitudes for cathodal stimulation [59]. Polarity-specific changes have also been observed for ERPs in the somatosensory modality [60-62], and for motor cortex excitability [63]. Furthermore, previous studies have shown that cathodal tDCS over the visual cortex decreases beta and gamma activity in response to visual stimulation [64], whereas cathodal tDCS over the motor cortex increases resting state theta and delta oscillation [5]. However, the study of oscillatory brain activity in the context of tDCS-induced WM alterations addresses a research question that has been under-investigated so far. Given that electrical brain stimulation can be a useful therapeutic tool for the treatment of cognitive deficits [65], a better understanding of tDCS-induced effects on the underlying brain activity seems to be of clinical relevance. In the present study we show that tDCS over the left DLPFC modulates theta and alpha band activity during WM performance in a polarity-specific way. Oscillatory power in the theta and alpha range was increased after anodal stimulation, while it was decreased after cathodal tDCS.

### Oscillatory brain activity in the context of a WM task

Changes in oscillatory brain activity play an important role in the formation of perception and memory and thus are essential for higher cognitive functions [66,67]. Accordingly, WM representations seem to be sustained by oscillatory brain activity [68,69]. Indeed, WM operations have been related to oscillatory brain activity in multiple frequency bands, including the theta (4-8 Hz), alpha (8-12 Hz), and beta (12-30 Hz) range [44]. In particular the performance in visual n-back tasks has been specifically associated with alterations in event-related theta and alpha band activity [70]. In this regard alpha band activity is assumed to reflect a general inhibition of non-task relevant areas [71,72] and may index the degree of inhibition necessary during internally, as opposed to externally, directed attention [73]. In contrast, theta band activity has been associated with memory encoding and retrieval [74-77] and may thus be particularly related to the function of the central executive of the WM system [78-80]. In the context of a WM task, theta band activity seems to reflect the continuous maintenance and manipulation of information required during the performance of an n-back task [70]. Interestingly, a power increase has been previously shown for both the alpha and theta band as a function of practice in a WM task [81].

In sum, previous studies have shown that alterations of rhythmic activity, specifically in the theta and alpha range, are associated with proper WM performance. These oscillations increase with behavioural enhancement. In the present study we show that tDCS over the left DLPFC induces altered WM performance by modulating its underlying theta and alpha activity. This result further highlights the importance of the left DLPFC and the specific role of theta and alpha activity during WM performance. Even though this interpretation implies that the modulatory effects of tDCS on WM are specifically related to the responsiveness of the left DLPFC, it may be assumed that altered local cortical excitability in one part of the responsible network influences the whole neural network associated with WM functions beyond the site of stimulation. Indeed, widespread tDCS-induced changes in cortical activity have been demonstrated by a previous neuroimaging study [82]. Thus, it is likely that by influencing one component of the WM network, the electrical stimulation of the left DLPFC had an influence on the functioning of the entire WM system.

## Conclusions

The present investigation studied the impact of anodal, cathodal or sham tDCS over the left DLPFC on the oscillatory brain activity associated with higher-order cognitive processing. Our results show that tDCS can change the organized cortical activity associated with WM in concert with systematic alterations of WM performance. To our knowledge, this is the first study investigating the effects of tDCS on oscillatory brain activity in the context of a WM task. The results of the study will provide a better understanding of the neuromodulatory effects of tDCS and demonstrate its potential both at fostering knowledge on the functional significance of brain oscillations and for therapeutic application.

## Methods

### Participants

Sixteen university students (10 females, mean age 25 ± 2 years) participated in the tDCS study. All participants were consistent right-handers [83] and had no metallic implant and no history of neurological or psychiatric illness. An intelligence test [84] showed that IQ levels of all the participants were in or above the range of the norm (mean 115 ± 13). Each participant gave written, informed consent prior to the experiment. All procedures in the study were approved by the ethics committee of the University of Zurich. In an additional control experiment a separate sample of 16 participants (12 females, mean age 24 ± 4 years) was investigated to measure the repetition effect on WM task performance without tDCS. This control group undertook one experimental session and performed the same behavioral task twice but without receiving any tDCS-treatment during the 15 min break in between. Experimental setting as well as instruction to the participants of the control group was identical to the stimulation group, except that no tDCS was applied and no EEG data were recorded. IQ levels of the participants in the control group were similar to those in the stimulated group (mean 117 ± 14).

### Transcranial direct current stimulation (tDCS)

**Figure 4:**
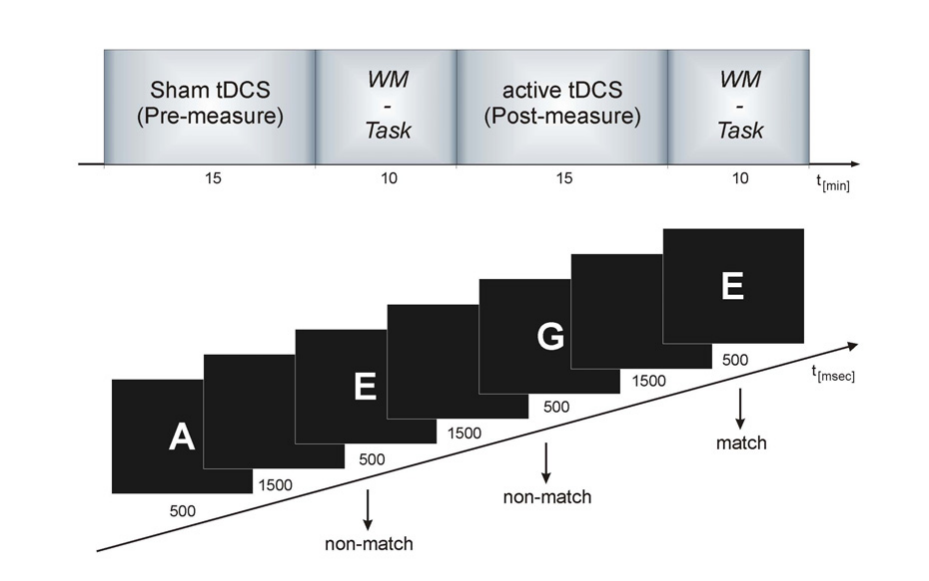
**Experimental design.** Top: After mounting EEG and tDCS electrodes, the experiment started with a 15-minute Sham-tDCS. For the next 10 minutes, participants performed a letter two-back working memory (WM) task while EEG was recorded. Subsequently, a 15 minute active-tDCS (anodal or cathodal) was applied, followed by a further 10-minute WM task. EEG recording was stopped during the stimulation periods. Each participant performed one anodal and one cathodal tDCS session separated by at least one day. The session order was counterbalanced across participants. An additional control group underwent one experimental session performing the similar WM task twice but without receiving a treatment during the 15 minute break in between. Bottom: Schematic description of the letter two-back working memory task.

The participants were seated comfortably in a recliner in front of a personal computer screen in an electromagnetically shielded room. TDCS was delivered by a battery-driven constant current stimulator (Eldith, NeuroCon GmbH, Germany) using a pair of rubber electrodes in a 5 × 7 cm saline-soaked synthetic sponge. For stimulation of the left DLPFC the active electrode (to which the term anodal/cathodal stimulation refers) was placed over F3 according to the International 10-20 system for electroencephalography (EEG) electrode placement [85]. The use of EEG electrode positions for DLPFC localisation has been applied before in studies using tDCS [8,26,29,52,86] and transcranial magnetic stimulation (TMS) [87]. Since both the left and right prefrontal cortex have been shown to subserve the WM system [41,88], and the left hemisphere seems to be particularly crucial for the processing of verbal WM tasks [42,43], we used an ipsilateral reference electrode over the left mastoid in order to avoid confounding biases arising from tDCS effects over the right hemisphere. In using this ipsilateral stimulation, we accepted the possibility that this mounting might reduce the tDCS-related effects on the underlying cortex, since it could cause current to be shunted to a greater degree through skin and cerebrospinal fluid. A constant current of 1.0 mA was applied for 15 min, with a linear fade in/fade out of 10 s. Each participant performed one anodal and one cathodal tDCS session separated by at least one day. The session order was counterbalanced across participants. Within each session, participants underwent one sham condition and one stimulation (i.e., anodal/cathodal) condition, and the sham condition always preceded the stimulation condition to avoid carry-over effects of tDCS (cf. Figure 4, top row). For the sham condition, the same electrode placement was used as in the stimulation condition, but the current was applied for 30 s, and was then ramped down without the subject's awareness. This procedure ensured that in both the sham and stimulation condition, participants experienced the initial itching that recedes over the first seconds of tDCS [89]. Accordingly, none of the participants was able to determine whether or not they received real or sham stimulation.

### Working memory assessment

After each sham and stimulation condition, participants performed a two-back letter working memory (WM) task with concurrent EEG recording. Stimulus presentation was controlled by the *Presentation *software (Neurobehavioral Systems, USA). Participants were stimulated with a sequence of white letters (A, B, C, D, E) which were presented on a black background in the centre of the screen. Each letter was presented for 500 ms with an interstimulus interval of 2 s. Each letter was followed by a white fixation cross that remained until the next letter was presented. A target letter was any letter repeated after one intervening letter. Participants were asked to respond to each letter as quickly and accurately as possible and to indicate whether the currently presented letter matched (left button) or did not match (right button) the letter which was presented two trials before (cf. Figure 4, bottom row). A brief practice sequence of 100 trials was given before the actual test. The test sequence consisted of 300 letters, with 77 matching letters (targets) and 223 non-matching letters (rejections).

### EEG recording and preprocessing

During the WM task, EEG was recorded continuously with thirty-two electrodes (Fp1, Fp2, F7, F3, Fz, F4, F8, FT7, FC3, FCz, FC4, FT8, T7, C3, Cz, C4, T8, TP9, TP7, CP3, CPz, CP4, TP8, TP10, P7, P3, Pz, P4, P8, O1, Oz, O2) located according to the International 10-20 system [85]. The electro-oculogram was recorded from two bipolar electrode pairs placed above and below the left eye, and on the outer canthi of each eye, respectively. A QuickAmp amplifier system (BrainProducts, Munich, Germany) was used for EEG recording. EEG data were recorded against the grand average reference and sampled at 500 Hz, and impedances were kept below 10 k Ω. EEG preprocessing and data analysis were carried out in *Brain Vision Analyzer *2.0 (BrainProducts, Munich, Germany), *EEGLAB *6.01 [90] and *FieldTrip *http://fieldtrip.fcdonders.nl/. EEG data were off-line treated with a 24 dB zero-phase Butterworth filter from 0.1 to 30 Hz and were segmented into epochs from -312 to 712 ms relative to stimulus onset. After baseline correction (-312 to 0 ms), epochs were automatically screened for unique and non-stereotyped artifacts using a probability function built into EEGLAB [91]. With this procedure, epochs that contained signal values exceeding three standard deviations were removed. Independent component analysis (ICA) was then applied to remove ocular artifacts [92,93]. After artifact removal, averages were computed for all remaining correct target responses (i.e., hits) for each of the four conditions (anodal, cathodal, sham preceding anodal (shamA), sham preceding cathodal (shamC)). In order to avoid multiple comparisons between neighbouring electrodes and to increase the signal-to-noise ratio, data from selected electrode sites (Pz, P3, P4, Oz, O1, O2) were pooled into an occipito-parietal ROI.

### Data Analysis

#### Behavioural data

To evaluate performance in the context of signal detection theory, d' was analyzed. As recently demonstrated, d' has the advantage to capture executive skills needed to perform n-back WM tasks without being influenced by demographic variables or IQ [94]. Values for d' were estimated for each subject by dividing the difference of Z(false alarms) and Z(hits) by the root mean square of 2. The discriminability measure d' captures the ability of the participant to discriminate between the two stimulus types, here the two-back target letters and the non-target letters. Additionally, reaction times (RT) for *Hits *and *False Alarms *(FA) were analyzed. To evaluate the impact of tDCS on the repetition-related increase in WM performance, we analyzed the pre-to-post measurements by computing the difference between the behavioural values assessed after active stimulation (anodal/cathodal) and the preceding sham stimulation (shamA/shamC), separately for the anodal (delta anodal, **Δ**A) and cathodal session (delta cathodal, **Δ**C). We compared these pre-to-post measurements, **Δ**A and **Δ**C, for each behavioural parameter using paired t-tests. Prior to this calculation, we evaluated the equality of the two sham conditions using paired t-test. This statistical analysis revealed no significant difference between the two sham conditions for d' (*t*_15 _= -0.34, *P *= 0.7). Similarly, RTs for *Hits *(*t*_15 _= 0.74, *P *= 0.5) and *False Alarms *(*t*_15 _= 0.27, *P *= 0.9) were not significantly different between the two sham conditions. To determine tDCS-induced modulations on the repetition-related increase in WM performance, we compared the pre-post differences (**Δ**A and **Δ**C) of the tDCS-treated group with pre-post measurements of a separate control group (**Δ**CG) that also performed the WM task twice but without receiving a tDCS treatment during the break in between (**Δ**A vs. **Δ**CG, and **Δ**C vs. **Δ**CG). Again, prior to this calculation, we evaluated the equality of the two experimental sham conditions of the tDCS-treated group and the pre-measurement (i.e., first repetition of the WM task) in the control group by means of independent-samples t-tests. This statistical analysis revealed no significant differences between the sham conditions of the tDCS-treated group and the pre-measurement of the control group (cf. Table 2).

**Table 2:** Comparison of pre-measurements

condition	measure	*t*	df	*P*
sham A vs. CG				
	d'	0.687	30	0.500
	RT-Hits	-0.910	30	0.371
	RT-FA	-0.528	30	0.602
sham C vs. CG				
	d'	1.080		0.290
	RT-Hits	-1.484	30	0.148
	RT-FA	-0.694	30	0.493

Table shows results of independent t-tests comparing the pre-measurements for d', RT-Hits (reaction time for Hits), and RT-FA (reaction time for False Alarms). Results show equivalence of pre-measures between the two experimental groups (shamA: sham condition preceding the anodal tDCS, shamC: sham condition preceding the cathodal tDCS) and control group (CG).

#### Analysis of event-related potentials

Event-related potentials (ERPs) were measured relative to the pre-stimulus baseline (-312 - 0 ms) for each condition (anodal, cathodal, sham preceding anodal (shamA), sham preceding cathodal (shamC)). Peak detection was performed for the occipito-parietal ROI (P1, N1 component) and for the Pz electrode (P3 component). This procedure entailed the detection of the most positive (P1, P3 component) or negative (N1 component) peaks within specific latency bands (P1: 80-140 ms; N1: 120-250 ms; P3: 230-500 ms). The time windows for peak analysis were defined on the basis of the global field power.

#### Analysis of event-related spectral perturbation

For each subject and each channel, event-related spectral perturbation (ERSP) was calculated using a wavelet-based analysis implemented in *Brain Vision Analyzer *2.0 software. We used a continuous wavelet transform (WT) with complex Morlet wavelets (morlet parameter c 3.8; 30 frequency steps from 1 to 30 Hz) to examine the frequency composition of single-trial epochs. The magnitudes of the WTs of single-trial epochs were then averaged to compute the total power of activity, which contains signal components that are phase-locked and non-phase-locked to the stimulus event. For each scale of the WT a baseline correction was applied by subtracting the mean amplitude within the -200 to -100 ms time window from each data point after stimulus onset. Similar to the procedures used in the ERP analysis, the data were subsequently pooled into an occipito-parietal ROI by averaging the ERSPs across different electrode sites (Pz, P3, P4, Oz, O1, O2).

TDCS effects on oscillatory brain activity were analyzed by computing ERSP differences between the separate conditions. For statistical comparisons, we used a nonparametric cluster-based randomization approach built into *FieldTrip*. This procedure defined clusters on the basis of the actual distribution of the data and tested the statistical significance of these clusters using a Monte-Carlo randomization method with correction for multiple comparisons [95]. The clustering used 500 randomizations and was performed in time and frequency simultaneously. The t-statistic of paired t-tests was calculated on a cluster-level by taking the sum of the t-values within the respective cluster.

## Authors' contributions

**TZ**: conceived of the study, designed the experimental paradigm, performed the statistical analysis and drafted the manuscript

**PS**: conceived of the study, performed the data acquisition and the statistical analysis and drafted the manuscript

**JDT**: performed statistical analysis and contributed to the manuscript

**LJ**: contributed to the hypothesis, and to the preparation of the manuscript

**CSH**: contributed to the design, discussion, and to the preparation of the manuscript

All authors read and approved the final manuscript.
